# A comparison of multiple sclerosis disease characteristics across three genetically diverse Asian racial groups in Singapore

**DOI:** 10.1038/s41598-024-65575-3

**Published:** 2024-06-26

**Authors:** Min Jie Koh, Seyed Ehsan Saffari, Janis Siew Noi Tye, Amelia Yun Yi Aw, Rachel Wan En Siew, Xuejuan Peng, Jeanne May May Tan, Kevin Tan, Tianrong Yeo

**Affiliations:** 1 Duke-NUS Medical School, 8 College Road, Singapore, 169857 Singapore; 2 Department of Neurology, National Neuroscience Institute, 11 Jalan Tan Tock Seng, Singapore, 308433 Singapore; 3 Lee Kong Chian School of Medicine, Nanyang Technological University, 11 Mandalay Road, Singapore, Singapore

**Keywords:** Demyelinating diseases, Multiple sclerosis

## Abstract

Studies in Western populations have shown that Black and Hispanic patients have an earlier age of Multiple Sclerosis (MS) onset and a more severe disease course characterised by faster disability accrual compared to Whites. It is yet unclear whether MS disease characteristics and clinical course differ amongst Asian racial groups. Singapore is uniquely poised to investigate this as its multi-racial population comprises three genetically diverse Asian racial groups—Chinese, Malay and South Asian. Herein, we sought to elucidate differences in the clinical phenotypes, disease-modifying therapy (DMT) usage, and disease course amongst these three Asian racial groups by performinga retrospective observational study on MS patients seen at the National Neuroscience Institute, Singapore. Data on demographics, disease characteristics, ancillary investigations, and DMT usage were collected. One hundred and eighty-eight patients were included (90 Chinese, 32 Malay, and 66 South Asian). Our findings showed that MS prevalence was the highest in South Asians followed by Malays and Chinese, while demographics, healthcare access, and longer-term disease course were identical across the racial groups. However, several differences and trends were elucidated: (1) South Asian patients had milder sentinel attacks (p = 0.006), (2) a higher proportion of Malay patients had enhancing lesions on their initial MRI (p = 0.057) and the lesion topography differed across the races (p = 0.034), and (3) more Malay patients switched out of their initial DMT (p = 0.051). In conclusion, MS disease characteristics were largely similar across these three Asian racial groups, and while there were some clinical and radiological differences at presentation, these did not influence longer-term outcomes.

## Introduction

While a high Multiple Sclerosis (MS) disease prevalence is present within Western populations, this is not uniform across the different racial groups. A recent large study in the United States showed that MS prevalence was highest in Whites, followed by Blacks, other non-Hispanic racial groups, and lowest in Hispanic individuals^1^, while studies from the UK demonstrated higher prevalence in Whites compared to Blacks and South Asians^2,3^. Beyond prevalence differences, it has been reported that MS disease characteristics differ across racial groups in the West. Black and Hispanic patients appear to have an earlier age of disease onset and a more severe disease course characterised by faster disability accrual compared to Whites^4–6^. Ancillary measures such as optical coherence tomography and MRI also reveal greater accelerated retinal damage and brain tissue loss in Blacks compared to Whites^7,8^.

To date, MS demographic and disease-related information in Asia have been mostly derived from countries that are largely racially homogenous, thus it is difficult to ascertain if there are differences in prevalence and disease characteristics between various Asian racial groups, especially those residing within the same geographical region (which may suggest shared environmental exposures). While MS prevalence is low in South-East Asia (~ 8 to 9 per 100,000)^9^, epidemiological studies from Singapore and Malaysia have offered some insights into MS prevalence within these multi-racial populations—prevalence is highest in Indians (South Asian ancestry), followed by Malays (Austronesian/Austroasiatic ancestry), and lowest in Chinese (East Asian ancestry)^10,11^. However, it is still unclear if there are differences in MS disease characteristics amongst these geographically proximate, yet genetically diverse Asian racial groups^12^. Singapore is uniquely poised to investigate this given its multi-racial population comprises three genetically distinct (demonstrated on a large-scale whole genome sequencing study in Singapore) Asian racial groups—Chinese, Malay, and South Asian, residing in a small island city state^12^. In this study, we sought to compare Chinese, Malay, and South Asian MS patients in Singapore with regards to their disease characteristics; specifically to use race as an exploratory variable to: (1) explore differences in MS phenotypes during initial presentation, (2) identify disparities in healthcare access and disease-modifying therapy (DMT) usage, and (3) highlight distinctions in disease severity and longer-term disability between these three Asian racial groups.

## Methods

### Study population

This retrospective observational study was performed at the National Neuroscience Institute (Tan Tock Seng Hospital Campus), which sees the majority of MS patients in Singapore. Consented patients who fulfilled the 2017 McDonald criteria for MS and were on active follow-up (to ensure robust data capture) were included^13^. Race was determined by that indicated on each individual’s national registration identity card in Singapore which was self-identified; for this study, racial groups were defined as Chinese, Malays, and South Asians (individuals whose ancestry can be traced back to India, Bangladesh, Pakistan, and Sri Lanka). Patients were classified into these racial groups regardless of generation (e.g. first, second) or time since migration.

### Data collection

Chart review was performed using electronic medical records up till the most recent clinical entry, with a defined data collection cut-off date of 1st December 2022. Data collected included: demographics, initial presentation of MS (severity, clinical phenotype, MS type, investigations), DMT usage (DMT efficacy, time on DMT, DMT changes), and longer-term disease course (total number of relapses, disability severity).

DMTs were categorised into high and low-to-moderate efficacy treatments; high efficacy DMTs being Fingolimod, Natalizumab, Cladribine, Alemtuzumab, anti-CD20 therapies, and autologous haematopoietic stem cell transplantation, while all other DMTs were categorised as low-to-moderate efficacy^14^. For the few patients who were started on DMTs prior to a formal diagnosis of MS or had DMTs initiated for other rheumatological conditions prior to the onset of MS, the time interval from diagnosis to DMT initiation was taken as 0. The percentage time spent on DMTs was calculated with the denominator being the total MS duration from first symptom onset.

Three disability indices were used during data collection: EDMUS Grading Scale (EGS), Expanded Disability Status Scale (EDSS) and The Multiple Sclerosis Severity Score (MSSS). The EGS was utilised to determine the severity of the first attack; it is a simplified version of the EDSS and has been shown to have excellent correlation with the equivalent score in the EDSS^15,16^. The advantage of using the EGS in this instance was the simplicity to ascribe a score from physical examination notes in the clinical records, some of which dated back many years ago. The EDSS is an established and validated MS disability rating scale; this was readily obtained for all patients using their recent clinical records to measure current and longer-term disability^17^. EDSS 6.0 was taken as a disability milestone and was confirmed on two clinical visits with an interval of ≥ 3 months. The MSSS is a score of a patient’s EDSS adjusted for the total duration of MS ranked against a large pool of patients^18^; this was derived for every patient based on their most recent EDSS (≥ 3 months away from last relapse, if applicable) prior to 1st December 2022.

### Statistical analysis

All statistical analyses were performed using SAS software version 9.4 for Windows (Cary, NC: SAS Institute Inc) and GraphPad Prism (version 8, GraphPad Software, San Diego, California USA). To identify differences in MS characteristics, univariate analyses were done on continuous data using Kruskal–Wallis one-way analysis of variance followed by Dunn’s test for post-hoc analysis, while Fisher exact test was utilised for categorical data with post-hoc Bonferroni correction. Univariate Cox proportional-hazards regression was performed to analyse the time taken to reach EDSS 6.0 amongst the three racial groups. P-values were two-tailed and significance set at < 0.05.

### Ethical approval

All procedures followed were in accordance with the ethical standards of the responsible committee on human experimentation (institutional and national) and with the Helsinki Declaration of 1975, as revised in 2000. This study was performed with IRB approval (SingHealth CIRB 2007/813/A, 2022/2462). Informed consent was obtained from all participants.

## Results

### Demographics

A total of 219 patients were initially identified, comprising 90 Chinese, 32 Malays, 66 South Asians; 31 were classified as ‘Others’ (Eurasians/mixed race, Caucasians, Filipinos, Vietnamese, and Indonesians) and were excluded from analysis. The three main racial groups, i.e., Chinese, Malays, and South Asians (n = 188) were included for analysis.

Singapore’s population has an ethnic composition of 74.1% Chinese, 13.6% Malay, 9% Indian, and 3.3% ‘Others’^19^. We observed a higher number of South Asians compared to Malay patients in our cohort even though South Asians was the smallest racial group in Singapore. To compare the relative MS prevalence across the three racial groups in this cohort, we calculated pair-wise prevalence ratios adjusted for the current population statistics for each racial group^19^; Chinese/Malay was 0.52, Chinese/South Asian was 0.17, and Malay/South Asian was 0.32. This finding was in line with a recent local MS epidemiology study which observed that Indians had the highest prevalence of MS, followed by Malays and then Chinese^10^.

Consistent with the known demographic features of MS patients, the age of onset of MS in our cohort was in the late 20 s (median 28.0 years) and there was a strong female preponderance (82.4%) (Table 1). The majority of patients (83.1%) were non-smokers and concomitant autoimmune conditions were uncommon (8.5%). No differences in demographic variables were observed between the racial groups.

**Table 1:** Demographic, clinical and investigational information of the study cohort, stratified by racial group.

	Chinese (n = 90)	Malay (n = 32)	South Asian (n = 66)	Total (n = 188)	p-value
Age at onset in years, median (Q1, Q3)	28.0 (23.0, 36.0)	25.0 (22.5, 31.5)	27.0 (25.0, 33.0)	28.0 (24.0, 35.0)	0.673
Gender (%)					
Female	77 (85.6)	26 (81.3)	52 (78.8)	155 (82.4)	
Male	13 (14.4)	6 (18.8)	14 (21.2)	33 (17.6)	0.725
Smoking status at first visit (%)					
Smoker	10 (14.7)	6 (26.1)	8 (15.7)	24 (16.9)	
Non-smoker	58 (85.3)	17 (73.9)	43 (84.3)	118 (83.1)	0.641
Any other autoimmune conditions (%)					
Yes	6 (6.7)	1 (3.1)	9 (13.6)	16 (8.5)	
No	84 (93.3)	31 (96.9)	57 (86.4)	172 (91.5)	0.336
Initial MS type (%)					
RRMS	82 (91.1)	32 (100.0)	63 (95.5)	177 (94.1)	
PPMS	8 (8.9)	0 (0.0)	3 (4.5)	11 (5.9)	0.195
Phenotype of onset attack (%)^a^					
TM	34 (41.5)	11 (34.4)	35 (56.5)	80 (45.5)	
Infratentorial	21 (25.6)	11 (34.4)	7 (11.3)	39 (22.2)	
ON	18 (22.0)	5 (15.6)	12 (19.4)	35 (19.9)	
Supratentorial	2 (2.4)	0 (0.0)	1 (1.6)	3 (1.7)	
Multiple	7 (8.5)	5 (15.6)	7 (11.3)	19 (10.8)	0.230
Severity of first MS attack by EGS, median (Q1, Q3)^a^	3.0 (2.0, 3.0)*	3.0 (2.0, 3.0)^+^	2.0 (2.0, 3.0)*^+^	3.0 (2.0, 3.0)	*0.006*
Enhancing lesion on initial MRI (%)					
Yes	42 (63.6)	20 (87.0)	37 (77.1)	99 (82.8)	
No	24 (36.4)	3 (13.0)	10 (22.9)	37 (27.2)	0.057
Location of enhancing lesion on initial MRI (%)					
Brain	24 (36.4)	9 (39.1)	21 (44.7)	54 (39.7)	
Spine	6 (9.1)	9 (39.1)	8 (17.0)	23 (16.9)	
Both brain and spine	12 (18.2)	2 (8.7)	8 (17.0)	22 (16.2)	
No enhancing lesion	24 (36.4)	3 (13.0)	10 (21.3)	37 (27.2)	*0.034*^Ψ^
First MRI brain T2 lesion load (%)^b^					
High	19 (35.2)	9 (52.9)	13 (35.1)	41 (38.0)	
Intermediate	11 (20.4)	3 (17.6)	12 (32.4)	26 (24.1)	
Low	24 (44.4)	5 (29.4)	12 (32.4)	41 (38.0)	0.436
CSF-restricted OCB (%)					
Present	40 (69.0)	22 (88.0)	35 (81.4)	97 (77.0)	
Absent	18 (31.0)	3 (12.0)	8 (18.6)	29 (23.0)	0.139

For variables ‘Enhancing lesion on initial MRI’, ‘Location of enhancing lesion on initial MRI’, ‘First MRI brain T2 lesion load’, and ‘CSF-restricted OCB’, not all patients in the study population was included due to missing data either due from the procedure not being performed or unavailability of records. Percentages indicated in these variables were based on the denominator of those with available data.

*CSF* cerebrospinal fluid, *EGS* EDMUS grading scale, *MRI* magnetic resonance imaging, *OCB* oligoclonal bands, *PPMS* primary progressive multiple sclerosis, *RRMS* relapsing remitting multiple sclerosis, *TM* transverse myelitis.

^a^Within RRMS patients only (Chinese n = 82, Malays n = 32, South Asian n = 63) as most PPMS patients do not present with relapses.

^b^The number of lesions were manually counted using axial T2-weighted sequence and cross referenced with the neuroradiologist report. Low lesion load was defined as 1–5 lesions, intermediate as 6–10 lesions and high as > 10 lesions.

*Pairwise comparison of Chinese vs. South Asian, Dunn’s post-hoc p = 0.010.

^+^Pairwise comparison of Malays vs. South Asian, Dunn’s post-hoc p = 0.006.

^Ψ^Pairwise comparison of Chinese vs. Malays, Bonferroni correction p = 0.019.

Significant values are in italics.

### Healthcare access

As a surrogate measure of health-seeking behaviour and healthcare access, the number of relapses between first symptom onset and first medical consult for MS was tabulated within patients subsequently diagnosed with relapsing remitting MS (RRMS) (Table 2). The median number of relapses between onset and consult were comparable among the three races at 1.0, indicating that most patients sought medical consult during or soon after their first attack. This observation suggests strong health-seeking behaviour across the racial groups which is likely to be facilitated by the ease of access to neurological care in Singapore. Next, we examined the number of relapses between the first medical consult for MS and diagnosis; the median was 0.0 within the cohort with no differences between the races. This suggests that despite the low prevalence of MS in Singapore, we were diagnosing MS in a timely manner (i.e., after the first attack and before subsequent attacks) and also highlights the fact the most patients already fulfilled the McDonald criteria for MS at their sentinel attack. Expectedly, the number of relapses from first onset to diagnosis was not statistically different amongst the racial groups.

**Table 2:** Number of relapses between key timepoints at disease onset within RRMS patients.

	Chinese (n = 82)	Malay (n = 32)	South Asian (n = 63)	Total (n = 177)	p-value
Number of relapses between onset and first consult, median (Q1, Q3)	1.0 (1.0, 1.0)	1.0 (1.0, 1.0)	1.0 (1.0, 1.0)	1.0 (1.0, 1.0)	0.462
Number of relapses between onset and diagnosis, median (Q1, Q3)	1.0. (1.0, 2.0)	2.0 (1.0, 3.0)	2.0 (1.0, 2.0)	1.5 (1.0, 2.0)	0.442
Number of relapses between first consult and diagnosis, median (Q1, Q3)	0.0 (0.0, 1.0)	0.5 (0.0, 1.25)	0.0 (0.0, 1.0)	0.0 (0.0, 1.0)	0.569

*RRMS* relapsing remitting multiple sclerosis.

### Initial MS presentation and workup

**Figure 1:**
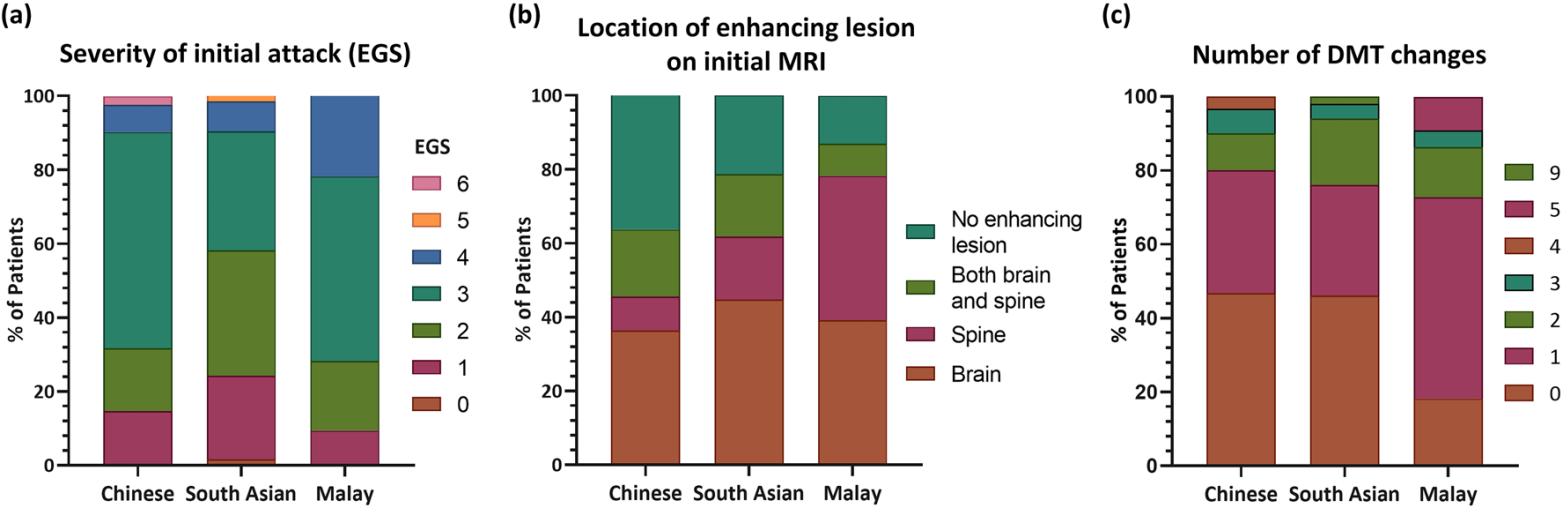
Stacked bar charts illustrating the comparative proportions between the three racial groups with regards to: (**a**) severity of initial attack within RRMS patients, (**b**) location of enhancing lesion(s) on initial MRI, and (**c**) number of DMT changes during MS disease course. *DMT* disease-modifying therapy; *EGS* EDMUS grading scale, *MRI* magnetic resonance imaging, *RRMS* relapsing remitting multiple sclerosis.

Table 1 details the various clinical and investigational parameters at MS onset. Within RRMS patients, we observed that the severity of the first attack was milder in South Asian patients compared to Chinese and Malay patients (median EGS; South Asian, 2.0 vs. Chinese, 3.0 vs. Malay, 3.0; p = 0.006) (Fig. 1A). An EGS of 2 represents minimal, not ambulation-related disability and able to run, whereas an EGS of 3 indicates significant, not ambulation-related disability or unlimited walking distance but unable to run^15,16^. There were no differences in the clinical phenotype of the first attack. Proportions of MS subtype (RRMS or primary progressive MS [PPMS]) were similar between the racial groups; the majority of the cohort had RRMS (94.1%) who presented predominantly with transverse myelitis (45.5%), followed by infratentorial syndromes (22.2%) and optic neuritis (19.9%).

We observed a trend in the proportions of patients who had gadolinium-enhancing lesions on initial MRI amongst the three racial groups, with Malays having the highest proportion (Malay, 87.0% vs. South Asian, 77.1% vs. Chinese, 63.6%; p = 0.057). Further stratification based on the topography of enhancing lesions revealed significant differences between the three groups (p = 0.034), particularly between Chinese and Malay patients (p = 0.019, Bonferroni correction), with a greater proportion of Malay patients having spinal cord restricted enhancing lesion(s) (39.1% vs. 9.1%) (Fig. 1B). There appeared to be more Malay patients (52.9%) with high (> 10) brain T2 lesion load on MRI compared to Chinese (35.2%) and South Asians (35.1%), while a lower proportion of Chinese patients had positive CSF-restricted oligoclonal bands (OCB) (69.0%) compared to Malays (88.0%) and South Asians (81.4%); however, these observations did not reach statistical significance.

### DMT usage

DMT usage (i.e. ever started on DMT), time taken to initiate DMT from MS diagnosis, and time on DMT (over MS disease duration) were similar between the comparative groups (Table 3). We observed a trend that fewer Malay patients commenced treatment with high efficacy DMTs compared to Chinese and South Asian patients (Malay, 18.2% vs. Chinese, 38.3% vs. South Asian, 34.7%; p = 0.248). Malay patients also had more frequent DMT changes (Fig. 1C); more Malay patients changed their DMT at least once as compared to Chinese and South Asian patients (Malay, 81.8% vs. Chinese, 53.3% vs. South Asian, 55.1%; p = 0.051).

**Table 3:** DMT uptake and usage pattern within RRMS patients.

	Chinese (n = 82)	Malay (n = 32)	South Asian (n = 63)	Total (n = 177)	p-value
DMT usage (%)					
Yes	60 (73.2)	22 (68.8)	49 (77.8)	131 (74.0)	
No	22 (26.8)	10 (31.3)	14 (22.2)	46 (26.0)	0.467
Efficacy of first DMT (%)					
High	23 (38.3)	4 (18.2)	17 (34.7)	44 (33.6)	
Low-to-moderate	37 (61.7)	18 (81.8)	32 (65.3)	87 (66.4)	0.248
Type of first DMT (%)					
Interferons	33 (55.5)	17 (77.3)	22 (44.9)	72 (55.0)	
Glatiramer acetate	1 (1.7)	–	2 (4.1)	3 (2.3)	
Teriflunomide	1 (1.7)	–	2 (4.1)	3 (2.3)	
Dimethyl fumarate	–	1 (4.5)	3 (6.1)	4 (3.1)	
Leflunomide	1 (1.7)	–	1 (2.0)	2 (1.5)	
Azathioprine	1 (1.7)	1 (4.5)	1 (2.0)	3 (2.3)	
Fingolimod	2 (3.3)	–	4 (8.2)	6 (4.6)	
Cladribine	6 (10.0)	–	5 (10.2)	11 (8.4)	
Alemtuzumab	1 (1.7)	3 (13.6)	1 (2.0)	5 (3.8)	
Natalizumab	5 (8.3)	–	1 (2.0)	6 (4.6)	
Rituximab	7 (11.7)	–	5 (10.2)	12 (9.2)	
Ocrelizumab	2 (3.3)	–	1 (2.0)	3 (2.3)	
Secukinumab	–	–	1 (2.0)	1 (0.8)	0.458
Time taken to initiate DMT from MS diagnosis in days, median (Q1, Q3)	241 (30.5, 975)	152 (28.5, 568)	121 (28, 527)	124 (29, 731)	0.535
Percentage time on DMT, median (Q1, Q3)					
High efficacy	36.4 (6.5, 74.2)	30.7 (0.0, 53.2)	26.5 (0.0, 59.1)	30.0 (0.3, 61.5)	0.225
Low-to-moderate efficacy	9.9 (0.0, 41.4)	19.9 (9.0, 58.3)	19.6 (0.0, 52.7)	16.6 (0.0, 48.1)	0.188
All DMTs	69.8 (46.1, 94.1)	58.0 (37.8, 86.5)	68.7 (31.9, 95.3)	65.8 (38.3, 93.1)	0.661
Number of DMT changes, median (Q1, Q3)	1.0 (0.0, 1.0)	1.0 (1.0, 2.0)	1.0 (0.0, 1.5)	1.0 (0.0, 1.0)	0.126
Switched out of initial DMT (%)					
Yes	32 (53.3)	18 (81.8)	27 (55.1)	70 (56.0)	
No	28 (46.7)	4 (18.2)	22 (44.9)	55 (44.0)	0.051

Table includes data within RRMS patients only as most PPMS patients were historically not commenced on DMTs.

*DMT* disease-modifying therapy, *PPMS* primary progressive multiple sclerosis, *RRMS* relapsing remitting multiple sclerosis.

### Longer-term disease course and disability

**Figure 2:**
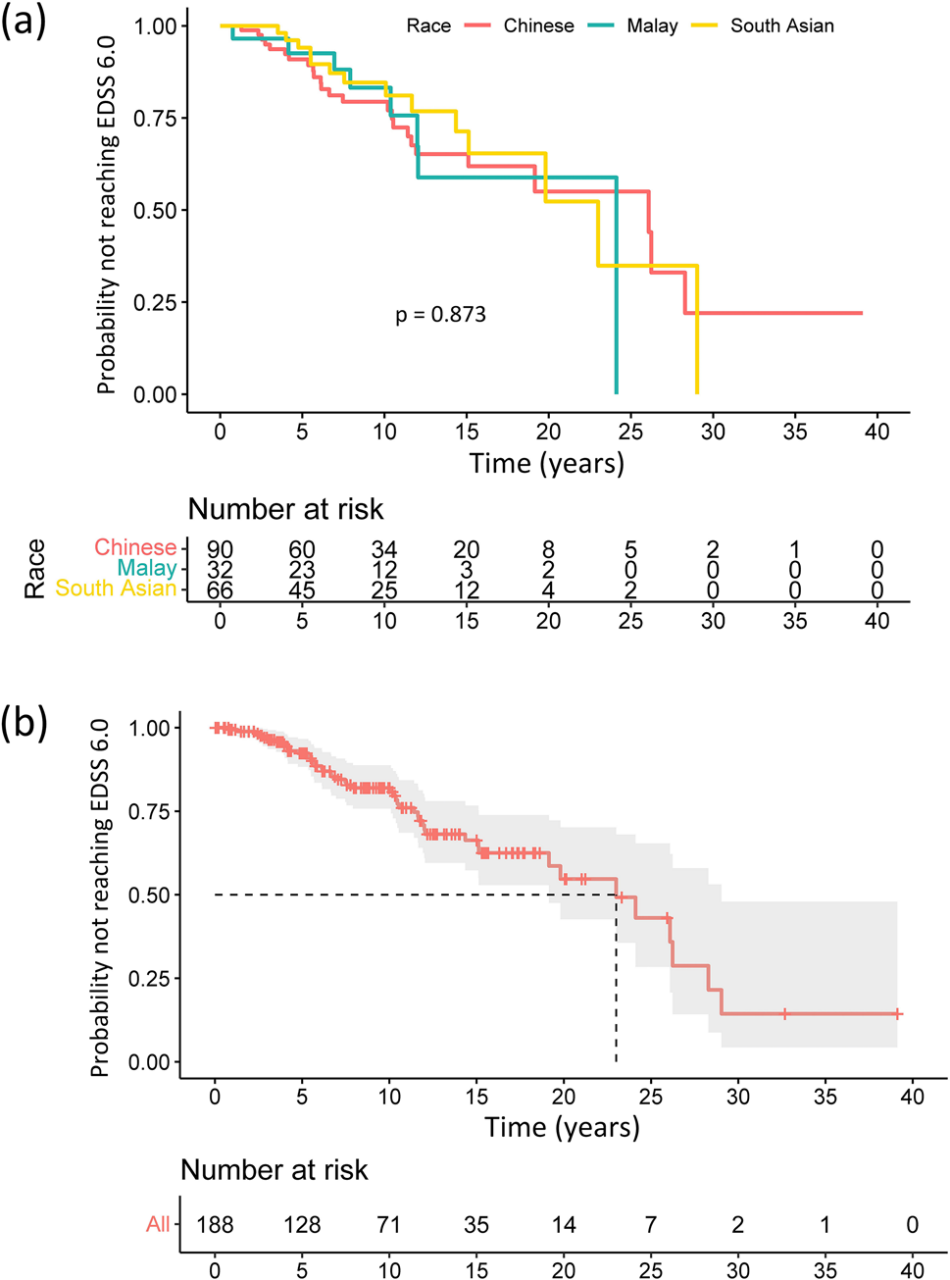
Kaplan–Meier curves showing time to reach EDSS 6.0 (**a**) stratified by race, and (**b**) of the entire study cohort. The median time to reach EDSS 6.0 was 23.0 years (dash line) for the entire cohort. *EDSS* expanded disability status scale.

The median MS disease duration among the racial groups were identical at around 10 years (Table 4). There were no significant differences amongst the racial groups with regards to progression and longer-term disability, conversion from RRMS to secondary progressive MS (SPMS), total number of relapses, proportion reaching EDSS 6.0, and latest MSSS (Table 4). Figure 2A shows the Kaplan–Meier curve for the time taken to reach EDSS 6.0; no differences were observed amongst the groups (log rank test, p = 0.873). The median time taken to reach EDSS 6.0 of the entire study cohort was 23.0 years (95% confidence interval 19.2–29.0) (Fig. 2B).

**Table 4:** Longer-term disability and disease severity of the study cohort.

	Chinese (n = 90)	Malay (n = 32)	South Asian (n = 66)	Total (n = 188)	p-value
Conversion to SPMS (from RRMS) (%)^a^					
Yes	17 (20.7)	6 (18.8)	10 (15.9)	33 (18.6)	
No	65 (79.3)	26 (81.3)	53 (84.1)	144 (81.4)	0.763
Total duration of MS in years, median (Q1, Q3)	10.1 (5.28, 15.7)	10.4 (6.83, 13.2)	9.30 (5.37, 13.0)	10.0 (5.56, 15.6)	0.641
Total number of relapses, median (Q1, Q3)^a^	3.0 (2.0, 5.0)	4.0 (2.0, 8.0)	3.0 (2.0, 5.0)	3.0 (2.0, 5.8)	0.407
Reached EDSS 6.0 (%)					
Yes	25 (27.8)	8 (25.0)	14 (21.2)	47 (25.0)	
No	65 (72.2)	24 (75.0)	52 (78.8)	141 (75.0)	0.646
MSSS, median (Q1, Q3)	2.3 (1.1, 6.6)	1.8 (0.7, 3.6)	2.4 (1.1, 4.8)	2.2 (1.0, 5.4)	0.495

*DMT* disease-modifying therapy, *EDSS* expanded disability status scale, *MSSS* multiple sclerosis severity score, *RRMS* relapsing remitting multiple sclerosis, *SPMS* secondary progressive multiple sclerosis.

^a^Analysed within RRMS only.

## Discussion

In this study, we compared Chinese, Malay, and South Asian MS patients in Singapore to identify potential differences in disease characteristics during initial presentation, healthcare access, DMT usage, and disease course. Consistent with our previous study, we found a clear difference in MS prevalence among the races (highest in South Asians, followed by Malays then Chinese). This is likely to be attributable to differential underlying genetic susceptibility given that these racial groups are genetically diverse (as demonstrated in a large whole-genome sequencing study of 4810 Singaporean Chinese, Malays, and Indians) and that environmental exposures are conceivably similar especially since Singapore is a densely populated, small island city state^12^. This difference in genetic susceptibility is suggested by human leucocyte antigen (HLA) haplotyping studies; a large study on bone marrow donors in Singapore showed that Indians have the highest frequency of the HLA-DRB1*15:01 allele (the HLA allele reported to have strongest effect on MS risk) while another study from India confirmed the association between MS with the HLA-DRB1*15:01 allele^20,21^.

Despite prevalence differences, the observed similarities in the other clinical features suggest that MS is by and large the same disease across these three Asian racial groups. We found that demographic characteristics and MS disease course were similar across the groups, save for a few minor differences at the initial presentation, namely, the milder severity of initial attacks in South Asian patients compared to Chinese and Malays, and to a certain extent, the proportions of patients with gadolinium-enhancing lesions (highest in Malays) and the location of these enhancing lesions on initial MRI. These differences, however, did not appear to influence longer-term disability between the racial groups. Of note, it is unlikely that the greater severity of the sentinel attack in Chinese and Malays was confounded by the higher rates of Neuromyelitis Optica Spectrum Disorder (NMOSD) in these racial groups with resultant diagnostic misclassification^10^. NMOSD is a well-recognised clinical entity in Singapore and cell-based assays for aquaporin-4 and myelin oligodendrocyte glycoprotein antibodies were available in our institution for ~ 15 years and ~ 5 years respectively.

The findings from our study also enabled us to juxtapose the MS disease characteristics of our Asian cohort (all three racial groups combined) with those reported in other populations. We found that most of these characteristics were generally similar to those reported in Western populations, consistent with a study by Kim et al. demonstrating that MS disease characteristics in South Korean patients showed no significant differences when compared to Canadian patients^22^. However, we wish to highlight several notable observations which will be discussed below.

Prevailing Western literature reports that 85–90% of individuals with MS have CSF-restricted OCB at baseline^23,24^. Whilst the majority of Malay and South Asian patients in our study had CSF-restricted OCB (~ 85%) at initial workup, this was only present in 69% of Chinese patients (although this difference was not statistically significant). Approximately 83% (68/82) of our Chinese RRMS patients had clinically definite MS (CDMS) (i.e. more than one clinical attack), and when we restricted our analysis to these Chinese CDMS patients, the proportion of those having positive CSF-restricted OCB remained low at 60% (27/45 who had CSF examination). This observation is in line with several studies reporting low CSF-restricted OCB positivity rate in Chinese patients^25,26^. The reason for this finding in Singaporean Chinese patients is unclear although it is possible that early MS disease at CSF sampling, lower latitude, and differential genetic susceptibility could be contributory^27–29^.

Three quarters of RRMS patients in our study cohort were commenced on DMTs, comparable to the high rates of DMT usage in Australia and Scandinavian countries^30^. Within those who were treated, one third were initiated on high efficacy DMTs. These observations are likely attributable to the access to a wide range of DMTs in Singapore including Rituximab biosimilars (often used as an off-label treatment). Furthermore, our institution has implemented the early use of high efficacy DMTs as current evidence points towards the association of this treatment strategy with lower long-term disability and likelihood of progressive disease^31,32^. We observed that a lower proportion of Malay patients were initiated on high efficacy DMTs, and consequently, a higher proportion switched out of their initial DMT which is likely due to breakthrough disease activity on lower efficacy treatments. There may be possible socio-behavioural factors affecting the uptake of high efficacy treatments amongst the racial groups in Singapore which merit further investigation.

It has been reported that approximately 50% of untreated RRMS patients convert to SPMS after 10–15 years from disease onset^33^. A more recent study by Fambiatos et al. observed a median time to SPMS of 32.4 years, taking into account the effect of DMTs (median proportion of time on treatment was 10.9%)^34^. The rate of secondary conversion in our study cohort was 18.6% after a median disease duration of 10 years which is comparable to that reported in the study by Fambiatos et al. (~ 10% after 10 years disease duration). Comparing disease severity between Asian and Caucasian patient populations, a previous study highlighted that MS disease course appear to be milder in Japanese patients compared to UK patients, with median MSSS of 3.34 and 5.87 respectively^35^. However, disparities were present between the two cohorts, in particular, the higher DMT usage in Japanese patients (83.7% vs. 17%). The median MSSS in our cohort was 2.2, comparable to recent studies in both Asian (South Korea: MSSS 1.77, mean disease duration 8 years) and Western cohorts (United States: MSSS 2.2, mean disease duration 20.1 years)^36,37^. We also calculated that the median time to reach EDSS 6.0 (from disease onset) of our study cohort to be 23.0 years, which is comparable to other contemporaneous population-based studies worldwide (Sweden, 30.4 years; Norway, 29.8 years; Wales 22.1 years)^38–40^. Older studies have reported a shorter time to EDSS 6.0 (France, 18.0 years; Canada 18.9 years) and it has been shown that the time taken to reach EDSS 6.0 from EDSS 1.0 was 18.5 years amongst patients in the placebo arms of randomised controlled trials^41–43^. These differences are likely to be explained by wider DMT usage as well as the use of more efficacious DMTs in the modern era^43^. Future local studies will explore if the rates of SPMS conversion, time to reach EDSS 6.0, and MSSS improve with more patients being initiated high efficacy treatments earlier in their disease course.

The main limitation of this study is the limited sample size of patients owing to the low prevalence of MS in Singapore. While several trends were noted, they were not statistically significant. We also ascribed patients into the defined racial groups regardless of generation or time since migration. This may be important for first generation patients as it is plausible that certain environmental factors may have already been acquired and modulated disease characteristics, prior to residing in Singapore. Despite these limitations, our study represents a novel attempt to comprehensively detail MS disease characteristics in three genetically distinct racial groups in Asia, and with this, enable comparisons to other MS cohorts globally.

## Conclusion

MS disease characteristics are largely similar across the main racial groups in Singapore and while there were some differences at initial presentation and in DMT use, these did not influence longer-term outcomes. Further studies encompassing more patients and the inclusion of sensitive disability indices would represent significant steps to deepening our understanding of MS among Asian races and with Western populations.

## Data Availability

Anonymised data can be made available to qualified investigators on reasonable request to the corresponding author.
